# Sequential treatment from bisphosphonate to denosumab improves lumbar spine bone mineral density in postmenopausal osteoporosis patients: A meta-analysis of randomized controlled trials

**DOI:** 10.1097/MD.0000000000040594

**Published:** 2024-11-15

**Authors:** Xu Jiang, Siyi Hou, Xiaolei Deng, Liyou Hu, Jian Wang, Decai Hou

**Affiliations:** a First Clinical College, Liaoning University of Traditional Chinese Medicine, Shenyang, Liaoning, China; b Department of Geriatrics, Affiliated Hospital of Liaoning University of Traditional Chinese Medicine, Shenyang, Liaoning, China; c Department of Orthopaedics II, Affiliated Hospital of Liaoning University of Traditional Chinese Medicine, Shenyang, Liaoning, China; d Graduate School, Liaoning University of Traditional Chinese Medicine, Shenyang, Liaoning, China.

**Keywords:** bisphosphonates, denosumab, meta-analysis, osteoporosis, postmenopausal, sequential treatment

## Abstract

**Background:**

Bisphosphonates are effective in the treatment of postmenopausal osteoporosis. However, their prolonged use induces adverse events and may lead to a rapid decline in bone mineral density (BMD) after discontinuation. Denosumab, a human monoclonal antibody, is a widely used antiresorptive agent that is more effective than bisphosphonates in improving bone density. Whether sequential treatment with denosumab after bisphosphonate therapy can maintain or further increase BMD at all sites has not been conclusively demonstrated. Thus, we performed a meta-analysis of randomized controlled trials (RCTs) to assess the effects of this sequential therapy on BMD.

**Methods:**

We searched the PubMed, Embase, and Cochrane Library databases from December 1, 1986, to May 2, 2024, for all RCTs that assessed the efficacy of sequential therapy of bisphosphonate transition to denosumab in postmenopausal women with osteoporosis. BMD changes at the lumbar spine, femoral neck, and total hip were used as outcomes. We assessed methodological quality, extracted relevant data according to the *Cochrane Handbook for Systematic Reviews of Interventions*, applied random-effects models for meta-analyses, performed heterogeneity analyses, and assessed publication bias.

**Results:**

A total of 3290 patients from 4 RCTs were included in the meta-analysis. Forest plot analysis showed that sequential treatment with bisphosphonate–denosumab was associated with higher lumbar spine BMD gain than continuous bisphosphonate treatment [mean difference (MD) = 5.50, 95% confidence interval (CI) = 5.26–5.75, *I*^2^ = 32.88%). No risk of bias was observed for the 4 trials, but there was an increase in femoral neck and total hip BMD. Moreover, analyses could not be performed because of high heterogeneity (femoral neck BMD: MD = 3.85, 95% CI = 2.84–4.85, *I*^2^ = 97.88%; total hip BMD: MD = 5.65, 95% CI = 4.28–7.02, *I*^2^ = 97.91%).

**Conclusion:**

Sequential therapy that involves a transition from bisphosphonates to denosumab had a positive effect on lumbar spine bone density, and this type of therapy may be a potential treatment option for increasing lumbar spine bone density in postmenopausal women.

## 1. Introduction

Osteoporosis is an insidious disease characterized by low bone mass and microarchitectural damage,^[1]^ and postmenopausal women are a major risk group, with approximately 62% of postmenopausal women suffering from osteoporosis.^[2]^ Fragility fractures due to osteoporosis have become a major public health threat owing to their significant morbidity, mortality, and healthcare burden.^[3]^ Vertebral fractures are the most common type of fragility fractures. However, given that back pain, height shortening, and other symptoms can be dismissed as aging-related symptoms, the symptoms of vertebral fractures are often ignored. Furthermore, its low diagnosis and treatment rate greatly reduces the quality of life of patients and increases the risk of death.^[4]^ Therefore, the prevention and control of osteoporosis in postmenopausal women are particularly important. The guidelines state that bone loss can be prevented through adequate calcium and vitamin D3 intake, smoking and alcohol cessation, and exercise therapy and recommend that medication should be administered for patients with reduced bone mass or low bone mass and a history of fragility fractures of the hip or spine.^[5]^ Some scholars have suggested that menopause-related problems such as osteoporosis can be mitigated by delaying or prolonging the childbearing age of women.^[6–8]^

Bisphosphonates are anti-bone resorption drugs that selectively inhibit the activity of farnesyl pyrophosphate synthase in osteoclasts, thereby inhibiting bone resorption and bone turnover to increase bone density and bone strength; these drugs are currently the mainstay of treatment for postmenopausal osteoporosis.^[9,10]^ However, long-term bisphosphonate treatment may incur a series of adverse events such as atypical femur fractures, osteonecrosis of the jaw, and renal damage.^[11–14]^ Although the incidence of adverse events is extremely low and has minimal effect on the therapeutic benefit,^[15]^ when considering drug discontinuation (i.e. “drug holiday”), adverse events should still be taken into account in conjunction with the long-term efficacy of bisphosphonates, risk of adverse events, and risk of fractures.^[16]^

Some studies have found that postmenopausal women with osteoporosis on long-term bisphosphonate therapy have reduced systemic bone mineral density (BMD) after drug discontinuation,^[17,18]^ and the American Society for Bone and Mineral Research recommends that postmenopausal women at high risk of osteoporosis should be maintained on bisphosphonate therapy or sequential anti-osteoporosis treatments after receiving bisphosphonate therapy for 3 to 5 years.^[19]^ Denosumab, a human monoclonal antibody, is another widely used anti-bone resorption drug. Unlike bisphosphonates, denosumab inhibits bone resorption by blocking the binding of RANKL to its receptor RANK and inhibiting osteoclast formation, activity, and survival.^[20]^ Studies have shown that denosumab is more effective than bisphosphonates in boosting bone density in patients with osteoporosis.^[21,22]^ Sequential therapy with bisphosphonate and transition to denosumab is superior to continuous bisphosphonate therapy for BMD improvement in postmenopausal women at a high risk of fracture.^[23]^ However, there are no studies that have generalized, analyzed, summarized, and drawn definitive conclusions from randomized controlled trials (RCTs) on this sequential therapy. Therefore, we conducted a meta-analysis of RCTs of sequential bisphosphonate therapy with transition to denosumab and continuous bisphosphonate therapy to fully assess the effect of this therapy on BMD in patients with postmenopausal osteoporosis.

## 2. Methods

This meta-analysis was conducted in accordance with the Preferred Reporting Items for Systematic Evaluation and Meta-Analyses (PRISMA) statement^[24]^ and registered with PROSPERO (CRD42024548514).

### 2.1. Search strategy

To search all articles in the PubMed, Embase, and Cochrane Library databases from December 1, 1986, to May 2, 2024, we used the following keywords “Osteoporosis, Postmenopausal” AND (“Bisphosphonates” OR “Alendronate” OR “Ibandronic Acid” OR “Risedronic Acid” OR “Zoledronic Acid” OR “Denosumab”) AND “Randomised Controlled Trial.” A summary of the search process is shown in Additional File 1. Two authors (LH and JW) independently searched the literature in duplicate without language restrictions. The final search was conducted on May 22, 2024.

### 2.2. Study selection

Two authors (LH and JW) independently selected and reviewed articles. Titles and abstracts were filtered based on their relevance to the topic. After reading the abstracts, the full texts were screened to select articles for inclusion in the meta-analyses. Articles eligible for inclusion were selected independently by both authors. If it was unclear whether an article should be included, it was discussed with a third author (DH) to reach a consensus.

The inclusion criteria were as follows: population: postmenopausal osteoporosis women were defined as postmenopausal women aged > 55 years and at high risk of fracture; intervention: sequential bisphosphonate-to-denosumab transition therapy, including a period of bisphosphonate discontinuation followed by denosumab and receipt of sequential bisphosphonate–denosumab therapy; control: treatment with bisphosphonate medication only, regardless of whether the medication was discontinued during the study period, with no restriction on specific types of bisphosphonate drugs; outcome indicators: at least 1 of the following outcomes was reported: changes in BMD at the lumbar spine, femoral neck, or total hip, and BMD should be measured by dual-energy X-ray absorptiometry; and study design: RCTs. During the discontinuation period, none of the participants received treatment other than basal therapy (oral calcium with vitamin D).

Patients with secondary osteoporosis combined with chronic kidney disease, malignancies, or other known metabolic bone diseases were excluded. Case-control studies, cohort studies, case series, trials with a non-randomized design, duplicate reports, trials with insufficient information, and trials with sample sizes of <100 patients were also excluded.

### 2.3. Data extraction

Two authors (JX and SH) independently extracted data from all eligible publications and carefully reviewed their counterpart’s extracted data. Any disagreements were resolved by a third author (DH). The following data were extracted: information related to references (authors and year of publication), study design (intervention design, subgroups, sample size, analysis, or treatment intent for each protocol), treatment (type of treatment, dosage, route of administration, and duration of follow-up), and outcomes (mean percentage increase in BMD at each site from baseline). If raw data were not provided in the paper, they were obtained from the original line graphs using AUTOMERIS software. Authors of individual papers were contacted if any information was missing.

### 2.4. Risk of bias assessment

Two authors (XD and LH) independently assessed the risk of bias using the Cochrane risk of bias tool^[25]^ in accordance with the PRISMA statement and used RevMan 5.3 for image production. Seven bias categories were assigned: random sequence generation (selection bias), allocation concealment (selection bias), blinding of participants and staff (performance bias), blinding of outcome assessment (detection bias), incomplete outcome data (attrition bias), and selective reporting (reporting bias). Each category consists of 3 levels: low, unclear, and high risk of bias. Disagreements were resolved by a third author (DH).

### 2.5. Statistical analysis

Data were combined using the mean difference and 95% confidence intervals (CIs). The heterogeneity of results across studies was assessed using Cochran *Q* statistic and the *I*^2^ statistic (*I*^2^ > 50% indicates significant heterogeneity).^[26]^ A meta-analysis was performed using a fixed-effects model, but a random-effects model was used in cases of significant heterogeneity.^[27]^ Publication bias was assessed using funnel and Galbraith plots. All statistical analyses were performed using Stata 17.0.

### 2.6. Ethical approval

All analyses were based on previously published studies; therefore, ethical approval and patient consent were not required.

## 3. Results

### 3.1. Search results

A total of 2511 articles were retrieved from PubMed, Embase, and the Cochrane Library, and 1500 articles were excluded because of duplication. After screening the titles and abstracts, another 964 irrelevant articles were excluded.

**Figure 1.**
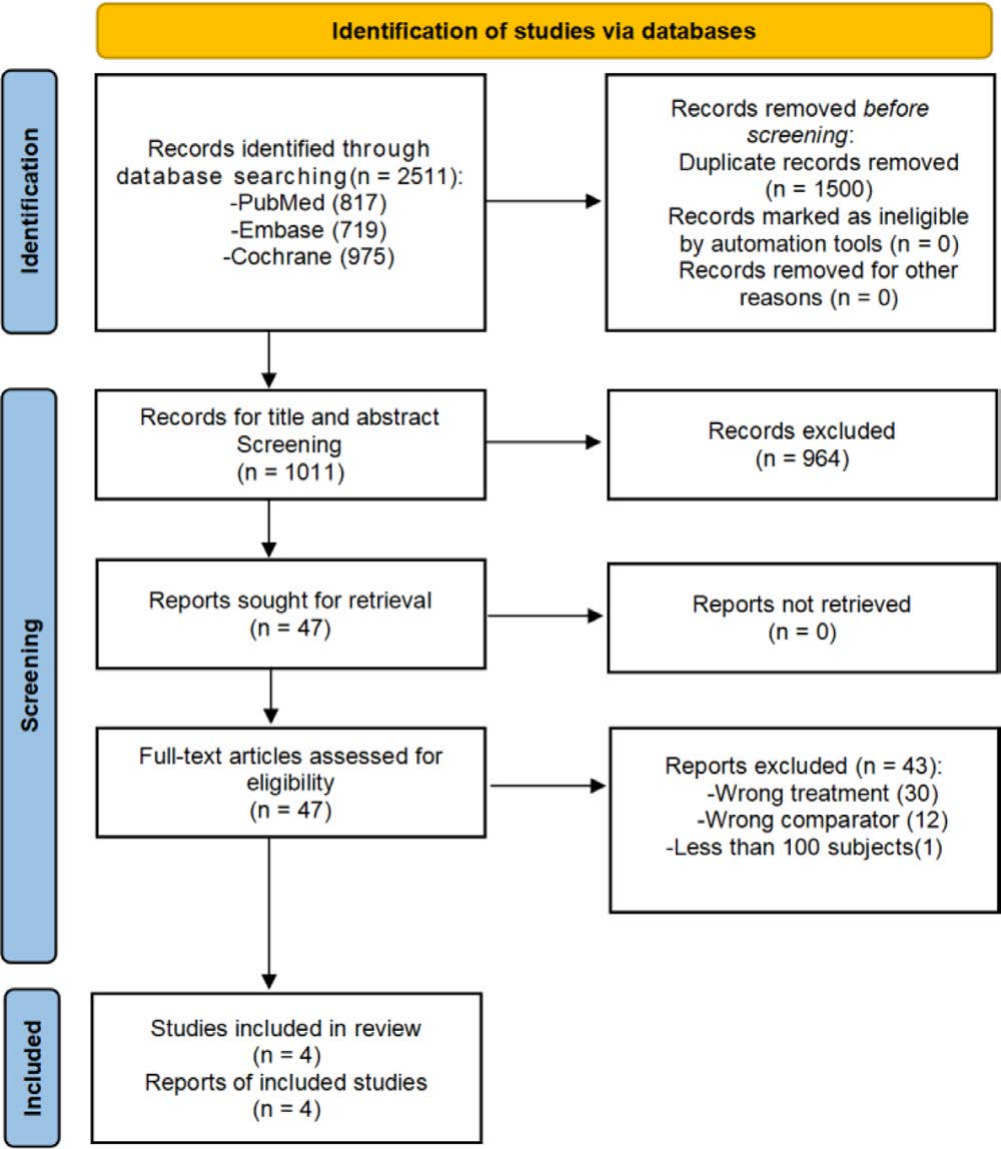
Flow diagram of the literature selection process.

After screening through the inclusion and exclusion criteria, 5 articles met the criteria for the next assessment, but 1 article^[28]^ was excluded because of a small sample size. Ultimately, 4 articles^[29–32]^ with a combined total of 3290 subjects were included in this study. The screening steps and reasons for exclusion are shown in Figure 1.

### 3.2. Study characteristics

The main characteristics of the included studies are summarized in Table 1. The total sample size of these trials was >600. The treatment duration after conversion was 12 months in all patients, and all patients received daily oral calcium and vitamin D supplements as adjuncts to therapy.

**Table 1:** Summary of included studies.

Authors (yr)	Age (yr), mean (sd)	Number of subjects	Basic intervention	First stage	Average time (mo)	Second stage	Average time (mo)
Brown 2014^[29]^	67.5 (7.6)	469	Calcium (≥ 500 mg/d) and vitamin D (800 IU/d)	Bisphosphonate therapy	12	60 mg denosumab/6 mo	12
	67.0 (7.3)	469		Bisphosphonate therapy	12	Continue bisphosphonate therapy	12
Miller 2016^[30]^	69.5 (7.7)	322	Calcium (1000 mg/d) and vitamin D (800 IU/d)	Bisphosphonate therapy	74.4	60 mg denosumab/6 mo	12
	68.5 (7.1)	321		Bisphosphonate therapy	76.8	Zoledronate 5 mg	12
Recknor 2012^[31]^	67.2 (8.1)	417	Calcium (≥500 mg/d) and vitamin D (≥800 IU/d)	Bisphosphonate therapy	16.7	60 mg denosumab/6 mo	12
	66.2 (7.8)	416		Bisphosphonate therapy	16.8	150 mg ibandronate/mo	12
Roux 2014^[32]^	67.8 (7.0)	435	Calcium (1000 mg/d) and vitamin D (800 IU/d)	Alendronate therapy	20.0	60 mg denosumab/6 mo	12
	67.7 (6.8)	435		Alendronate therapy	27.2	150 mg risedronate/mo	12

**Figure 2.**
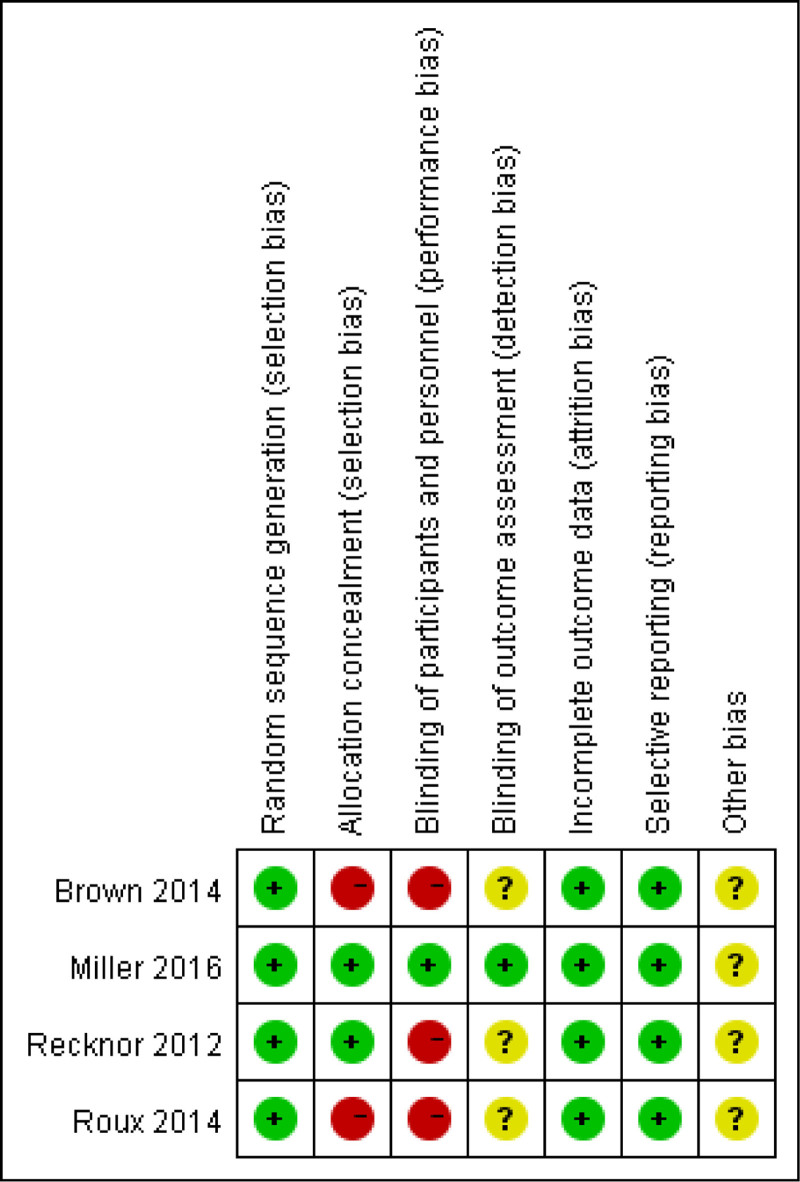
Risk of bias graph and risk of bias summary. “+” means low risk, “?” means unclear risk, and “−” means high risk.

**Figure 3.**
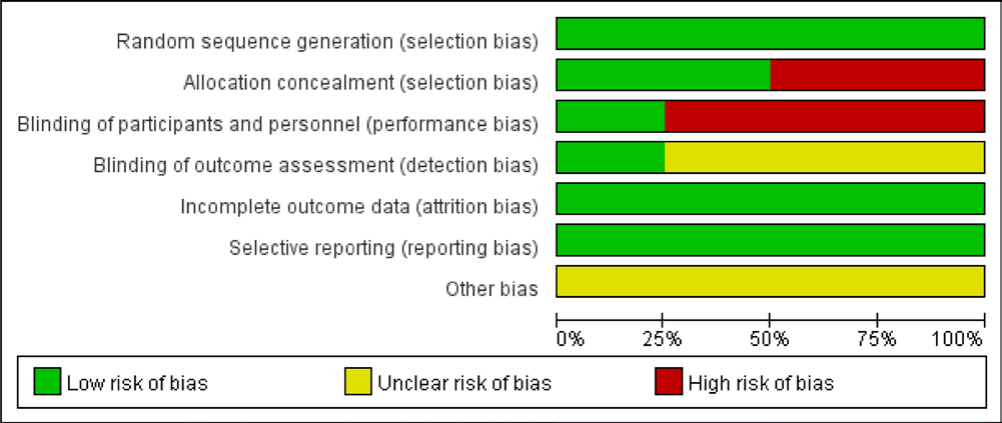
Risk of bias summary.

The details of the risk of bias are summarized in Figures 2 and 3. Randomized sequence generation was adequately reported in all trials. Allocation concealment was adequately reported in 2 trials^[30,31]^ but not in the remaining trials.^[29,32]^ Three trials were open-label,^[29,31,32]^ which may have contributed to performance bias. Blinding of the outcome assessment was adequately reported in 1 trial^[30]^ and unclear in 3 trials.^[30–32]^ Information from all trials was insufficient to assess the presence of other biases.

#### 3.2.1. Effect of bisphosphonate–denosumab sequential therapy on lumbar spine BMD:

**Figure 4.**
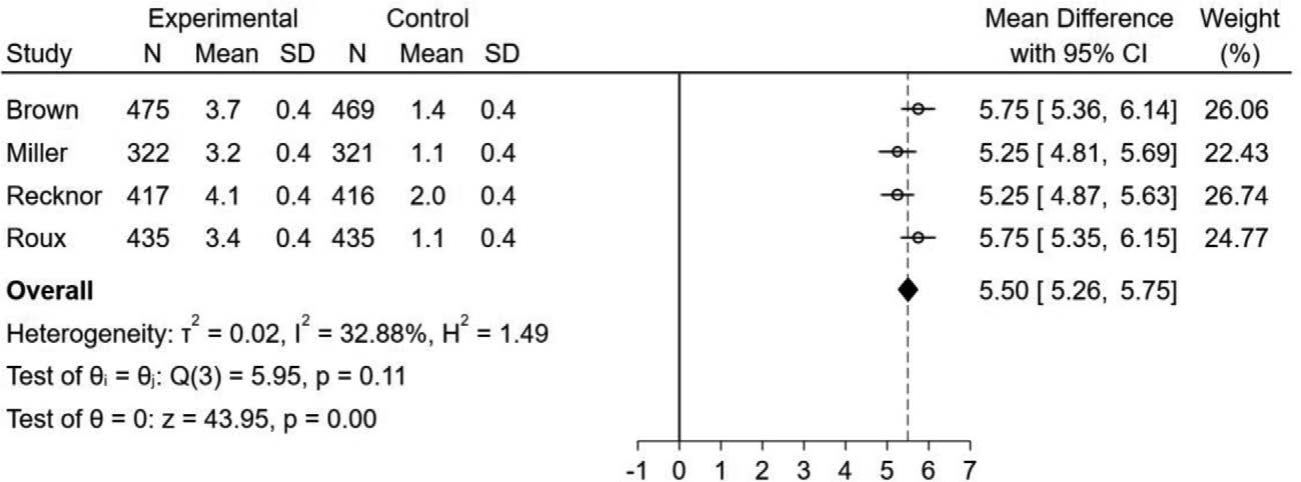
Forest plot for the percentage increase in lumbar spine bone mineral density (BMD) from baseline.

The forest plot (Fig. 4) showed that bisphosphonate–denosumab sequential therapy had a positive significance on increasing lumbar spine BMD (MD = 5.50, 95% CI = 5.26–5.75, *I*^2^ = 32.88%), with low heterogeneity among the studies.

#### 3.2.2. Effect of bisphosphonate–denosumab sequential therapy on femoral neck BMD:

**Figure 5.**
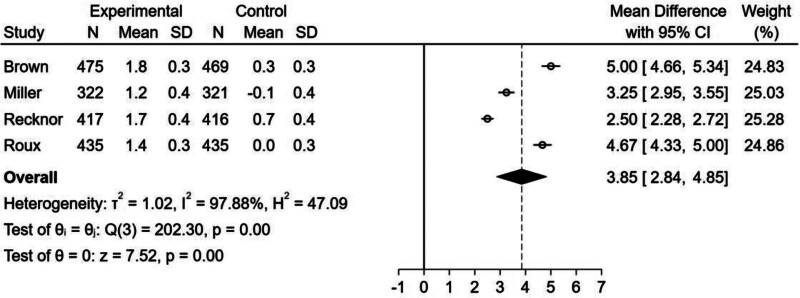
Forest plot for the percentage increase in femoral neck BMD from baseline.

The forest plot (Fig. 5) showed that there was an increase in femoral neck BMD in the bisphosphonate–denosumab sequential therapy group (MD = 3.85, 95% CI = 2.84–4.85, *I*^2^ = 97.88%). However, there was statistically significant heterogeneity among studies, and we were unable to draw a conclusion because the source of heterogeneity could not be identified through subgroup and sensitivity analyses.

#### 3.2.3. Effect of bisphosphonate–denosumab sequential therapy on total hip BMD:

**Figure 6.**
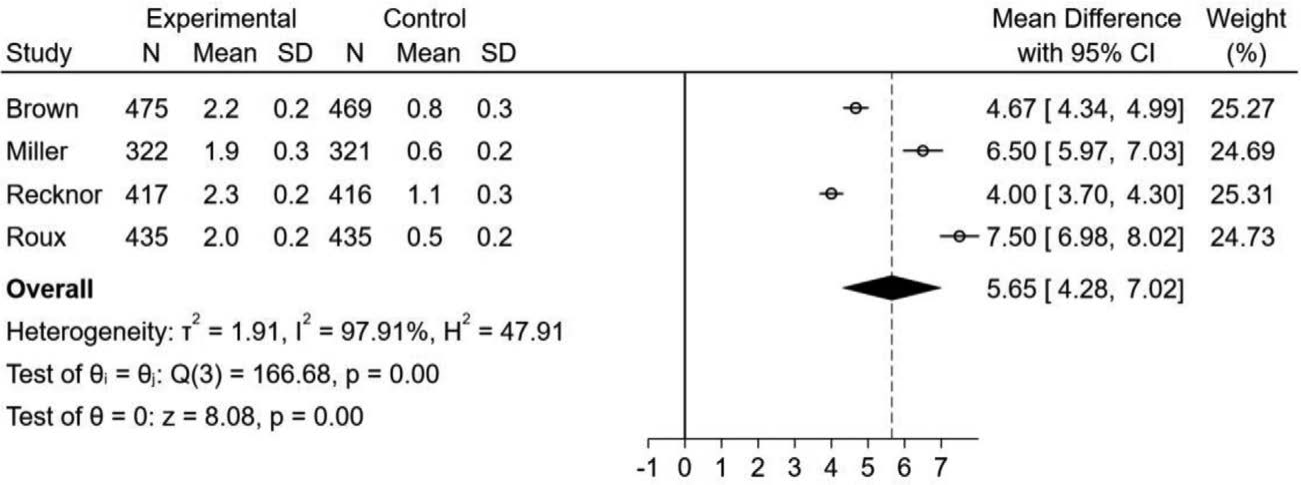
Forest plot for the percentage increase in total hip BMD from baseline.

Similar to the femoral neck index, although the forest plot (Fig. 6) showed an increase in the percentage of total hip BMD in the bisphosphonate–denosumab sequential therapy group (MD = 5.65, 95% CI = 4.28–7.02, *I*^2^ = 97.91%), there was significant heterogeneity among the studies, and we were unable to find the source of heterogeneity through subgroup and sensitivity analyses. Therefore, we were unable to draw any conclusions.

### 3.3. Publication bias

**Figure 7.**
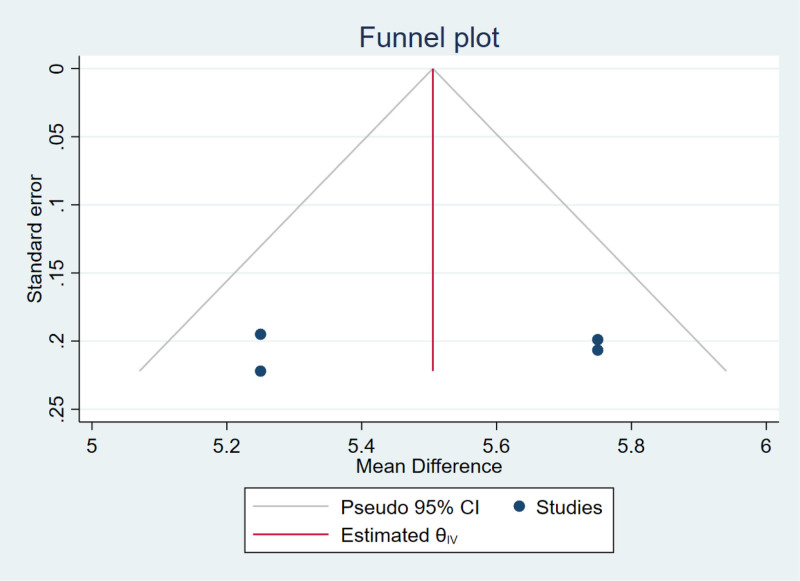
Funnel plot for publication bias in the percentage increase in lumbar spine BMD.

**Figure 8.**
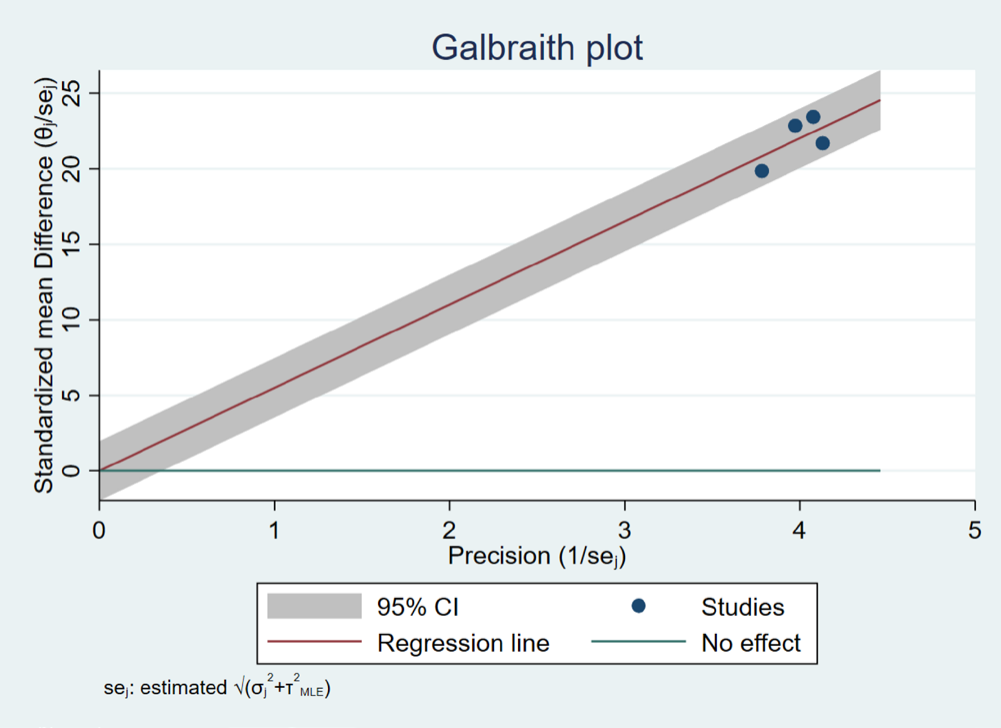
Galbraith plot for percentage increase in lumbar spine BMD.

Publication bias in lumbar spine BMD was assessed using funnel and Galbraith plots. The approximately symmetrical funnel plot (Fig. 7) revealed no significant publication bias. The Galbraith plot (Fig. 8) results for lumbar spine BMD showed no significant heterogeneity across studies. We were unable to determine the source of heterogeneity of femoral neck and total hip BMD changes from the Galbraith plots.

## 4. Discussion

This meta-analysis examined the transition of postmenopausal osteoporosis pharmacological treatment from bisphosphonates to sequential therapy with denosumab and ultimately included data from 4 trials with a total of 3290 subjects. Based on forest plots showing that sequential therapy with denosumab to bridge long-term bisphosphonate therapy was more effective than continuous bisphosphonate therapy in improving lumbar spine BMD but inconclusive for femoral neck and total hip BMD, we concluded that sequential denosumab therapy may be a more effective treatment strategy.

To the best of our knowledge, this is the first meta-analysis to assess the effectiveness of this sequential bisphosphonate transition to denosumab therapy, and the results of this study may be useful for improving lumbar spine bone density in postmenopausal women with osteoporosis.

Previous meta-analyses have only evaluated the effectiveness of bisphosphonates or denosumab alone for the treatment of osteoporosis. Several studies have compared the efficacy of various types of bisphosphonates in the treatment of osteoporosis with a placebo group,^[33–35]^ confirming the positive effects of bisphosphonates on osteoporosis, especially in postmenopausal women. Other studies have found an advantage of denosumab over placebo in increasing BMD,^[36,37]^ but the above studies were limited to a single type of drug therapy. Lou et al^[38]^ conducted a traditional meta-analysis of RCTs of sequential therapy with multiple drugs and found that sequential therapy increased BMD, however, during their study, it was broadly defined that all 2-phase therapeutic treatments with different strategies were defined as sequential therapy, including switching between different types of drugs and switching between single drugs and combinations. Moreover, the control group included placebo and single-type anti-osteoporosis drug treatment, which broadened the scope of the study but inevitably increased the risk of bias in the results. Han et al^[39]^ performed a net meta-analysis of drug-type switching in sequential therapy. The results revealed that switching from 1 antiresorptive agent to another had the most significant positive effect on BMD, which is consistent with our findings; however, their study was limited to comparing the inter-conversion between antiresorptive therapy, pro-osteosynthesis agent therapy, and the combination of medications, and they did not compare the specific therapeutic strategies in detail. In our study, we defined the experimental group as 1 phase of bisphosphonate therapy followed by another phase of denosumab therapy and the control group as continuous bisphosphonate therapy. We screened articles according to the above definitions to obtain reliable and accurate results.

In conclusion, sequential bisphosphonate–denosumab treatment was superior to sequential bisphosphonate treatment in improving lumbar spine bone density. We speculate that the reasons for this may be as follows, the mechanism of action of bisphosphonates is selective adhesion and retention in bone, binding and inhibition of farnesyl pyrophosphate synthase activity, and promotion of osteoblast apoptosis.^[40]^ Furthermore, these effects persist with cumulative bisphosphonate doses.^[41]^ Bisphosphonates are retained in the bone for a longer period, and drugs exposed to the bone surface dissociate and return to circulation and reattach to the bone to inhibit bone resorption.^[42]^ Denosumab prevents the maturation of osteoclast precursors and promotes osteoclast apoptosis by binding to the cytokine RANKL and preventing its binding to the RANK receptor.^[43]^ Bisphosphonates can be retained in the circulation for up to 10 years after discontinuation.^[42]^ In the sequential treatment group, denosumab injected after conversion therapy and circulating bisphosphonates acted simultaneously through 2 different mechanisms to promote osteoclast apoptosis and improve BMD. This method was superior to continuous bisphosphonate single-target therapy. Additionally, the results of several meta-analyses showed that denosumab, but not bisphosphonates, significantly increased BMD in patients with osteoporosis.^[21,22]^ The main reason for this finding may be related to the different mechanisms of action of these drugs at the tissue and basal levels. Furthermore, the greater accessibility of denosumab to cortical bone than bisphosphonates, which are more permeable in the bone matrix, provides greater benefit in dual-energy X-ray absorptiometry^[44]^; therefore, denosumab improves BMD after switching therapies with the same duration of dosing compared with bisphosphonates. Our conclusion may be attributed to a combination of these reasons.

Although our study is the first meta-analysis comparing sequential therapy with bisphosphonate transition to denosumab with continuous bisphosphonate therapy, it has some limitations. First, only 4 RCTs were included in our meta-analysis, and although the sample size was 3290 patients, the small number of included studies may have reduced the confidence of the results. Second, although we used a random-effects model, the heterogeneity of the percentage increase in femoral neck (*I*^2^ = 97.88%) and total hip (*I*^2^ = 97.91%) BMD was too high, and we were unable to find the source of heterogeneity through subgroup and sensitivity analyses, even though the results of the forest plot showed that there was an increase in BMD at the femoral neck and total hip joint. Limited by the heterogeneity, we could not draw any conclusions for these 2 BMD areas. In terms of interventions, the type and measurement of bisphosphonate therapy before switching treatments were unclear in the included studies. In terms of outcome metrics, the included studies had a short follow-up period, with an intervention duration of only 12 months. There were no studies with long-term follow-up assessments and interventions; therefore, no conclusions on the long-term clinical effect could be drawn. Considering these limitations, we recommend a conservative approach for our conclusions. Therefore, more high-quality studies with accurate interventions of longer durations and follow-up times should be conducted to clarify the effectiveness of this treatment strategy.

## 5. Conclusion

Our study suggests that sequential therapy with bisphosphonates and denosumab has a positive effect on lumbar spine bone density in patients with postmenopausal osteoporosis and may be a potential therapeutic strategy.

## Acknowledgments

We would like to thank Editage (www.editage.cn) for the English language editing.

## Author contributions

**Conceptualization:** Xu Jiang.

**Data curation:** Xu Jiang, Siyi Hou, Liyou Hu, Jian Wang.

**Formal analysis:** Liyou Hu, Jian Wang.

**Funding acquisition:** Xiaolei Deng.

**Methodology:** Xu Jiang, Xiaolei Deng.

**Supervision:** Decai Hou.

**Validation:** Liyou Hu, Jian Wang, Decai Hou.

**Writing – original draft:** Xu Jiang, Siyi Hou.

**Writing – review & editing:** Xu Jiang, Siyi Hou, Xiaolei Deng.
